# The pre-metastatic niche in lymph nodes: formation and characteristics

**DOI:** 10.1007/s00018-021-03873-z

**Published:** 2021-07-09

**Authors:** Lionel Gillot, Louis Baudin, Loïc Rouaud, Frédéric Kridelka, Agnès Noël

**Affiliations:** 1 Laboratory of Tumor and Development Biology, GIGA-Cancer, Liege University, Avenue Hippocrate 13, 4000 Liege, Belgium; 2 Department of Obstetrics and Gynecology, CHU of Liege, 4000 Liege, Belgium

**Keywords:** Lymph node, Pre-metastatic niche, Extracellular matrix, Lymphangiogenesis, Metastasis

## Abstract

Lymph node metastasis is a crucial prognostic parameter in many different types of cancers and a gateway for further dissemination to distant organs. Prior to metastatic dissemination, the primary tumor prepares for the remodeling of the draining (sentinel) lymph node by secreting soluble factors or releasing extracellular vesicles that are transported by lymphatic vessels. These important changes occur before the appearance of the first metastatic cell and create what is known as a pre-metastatic niche giving rise to the subsequent survival and growth of metastatic cells. In this review, the lymph node structure, matrix composition and the emerging heterogeneity of cells forming it are described. Current knowledge of the major cellular and molecular processes associated with nodal pre-metastatic niche formation, including lymphangiogenesis, extracellular matrix remodeling, and immunosuppressive cell enlisting in lymph nodes are additionally summarized. Finally, future directions that research could possibly take and the clinical impact are discussed.

## Introduction

Many types of cancer, including melanoma, breast, oral, pancreatic and cervical cancers, disseminate through the lymphatic system [1–5]. In a large number of cases, lymph nodes (LNs) are relay the first metastases and the presence or absence of LN metastases is a crucial prognostic parameter for clinicians [6]. Indeed, the presence of tumor cells in the first draining LN, the so-called sentinel LN, is regarded as a predictor for poor patient outcome [7]. The expression of lymphangiogenic growth factors, high lymphatic vessel (LV) density, and high incidence of lymphovascular invasion are typically associated with LN metastases and poor patient outcome [8, 9]. Metastatic dissemination to LNs develops when tumor cells become detached from the primary neoplasm, enter an LV and are subsequently transported to the sentinel LN where they initially accumulate in the nodal subcapsular sinus (SCS). Within the LN, disseminated tumor cells may either be destroyed, pass through the LN and enter the efferent LV, or remain in the LN where they form a colony [10, 11]. It has been debated at length whether cancer cells in LNs can secondarily seed distant metastases and colonize in distant organs. LN metastases were either viewed as clinically inconsequential [12, 13] or had the potential to seed distant organs [14, 15]. Two elegant studies demonstrated the migration of metastatic cells from LNs to distant organs in pre-clinical models [16, 17]. These data provided a definitive proof-of-concept that metastatic cells in LNs can go on to seed distant organs. They also provide an indication that, when treating LN metastases, the aim should be, not only to obtain local control but also to prevent distant disease and, therefore, death. Nevertheless, there is still no explanation as to why some tumors tend to metastasize in LNs, while others intravasate directly into blood vessels and reach distal sites via the blood stream.

The concept of a pre-metastatic niche was first formulated by David Lyden and colleagues 15 years ago [18]. This pioneering study revealed that factors shed or secreted by tumor cells provide the microenvironment, within the organ, where metastases may later develop. These factors prepare the target organ to support the survival and proliferation of disseminating tumor cells. The main events in such a priming process include the secretion of pro-metastatic growth factors and chemokines/cytokines, as well as the release of extracellular vesicles (EVs) by the primary tumor. These primary tumor-derived factors induce the recruitment of specific cell types, an escalation in numbers of immunosuppressive cells and the remodeling of the extracellular matrix (ECM) in the pre-metastatic organ. These molecular and cellular changes create a unique microenvironment that will support subsequent metastatic growth [8, 10, 18–20]. Pre-metastatic niche formation has been described in detail for the lung [21], liver [22] and bone [23], with some specificities for each organ [24–26]. However, less is known about the pre-metastatic niche in LNs. Hirakawa et al. were the first to observe LN remodeling at a pre-metastatic stage in 2005 [27] and 2007 [28]. They proved that the vascular growth factors (VEGF-A and VEGF-C) are responsible for inducing lymphangiogenesis in sentinel LNs. Since then, a number of studies have elucidated a number of distinctive features of pre-metastatic LNs, including increased lymphangiogenesis and lymph flow, remodeling of high endothelial venules (HEVs), recruitment of myeloid cells and reduction of effector lymphocyte numbers and function[18, 28, 29]. This review will begin by describing the specific structure of LNs under physiological conditions to more clearly describe the tissue remodeling set in motion by the primary tumor. The latest findings on key components and mechanisms involved in pre-metastatic niche formation in LNs will also be summarized.

## Cellular composition and compartmentalization in LNs under physiological conditions

The lymphatic system is a unidirectional, blind-ended vascular network, of not only lymphatic capillaries and larger collecting vessels, but also secondary lymphoid organs such as LNs. This vascular system is essential for maintaining fluid homeostasis, absorbing dietary lipids and transporting immune cells and soluble antigens from peripheral tissues towards LNs and the central circulatory system [30, 31].

### LN development

LN formation during fetal development has been studied through the generation and phenotyping of various gene-deficient mice but is not yet fully understood [32–35]. However, what is known is that the interaction between lymphoid-tissue inducer (LTi) cells and lymphoid-tissue organizer (LTo) cells is crucial for LN development [36]. LTi cells arising in the fetal liver are attracted to LN development sites by a gradient of chemokines, including CXCL13, CCL19 and CCL21 [37]. In a mouse model, the loss of CXCR5, a receptor for CXCL13, prevented the formation of peripheral LNs [38], stromal LTo cells expressed lymphotoxin-β-receptor (LTβR), while LTi cells produced its ligand, lymphotoxin-α_1_β_2_. This interaction between the two cell types induced an upregulation of adhesion molecules. For instance, vascular cell adhesion molecule 1 (VCAM-1) promoted the retention of hematopoietic cells in forming LNs [39]. LTβR signaling induced the secretion of VEGF-C by LTo cells, which could potentially attract lymphatic endothelial cells (LECs) into the developing organ. LECs surrounded LTi and LTo clusters and express CCL21, which further drew in LTi cells and activated LECs [40]. This activation was attributed to the expression of the receptor activator of NF-κB (RANK) by LECs. Accordingly, the ablation of RANK expression in LECs blocked LTi organization and LN formation [41]. Collecting lymphatic vessels are required for the transport of LTi cells, the formation of the LN capsule and SCS specialization in embryonic stages. Indeed, SCS specialization coincides with lymphatic vascular maturation. LECs of the LN lymphatic cup are organized in a double layer. LECs of the outer layer expressed FOXC2 (a marker for collecting vessels), whereas those of the inner layer expressed LYVE1, ITGA2B and MADCAM, specific markers of LECs lining the floor (fLEC). The genetic loss of FOXC2 in LECs from embryos is characterized by the absence of valves as a result of the suspension of collecting vessel development. In those mice, LN capsule formation was impaired, and SCS LECs failed to express ITGA2B. These results demonstrated that FOXC2 ensures collecting vessel maturation and capsule specialization [38].

### LN organization

**Fig. 1:**
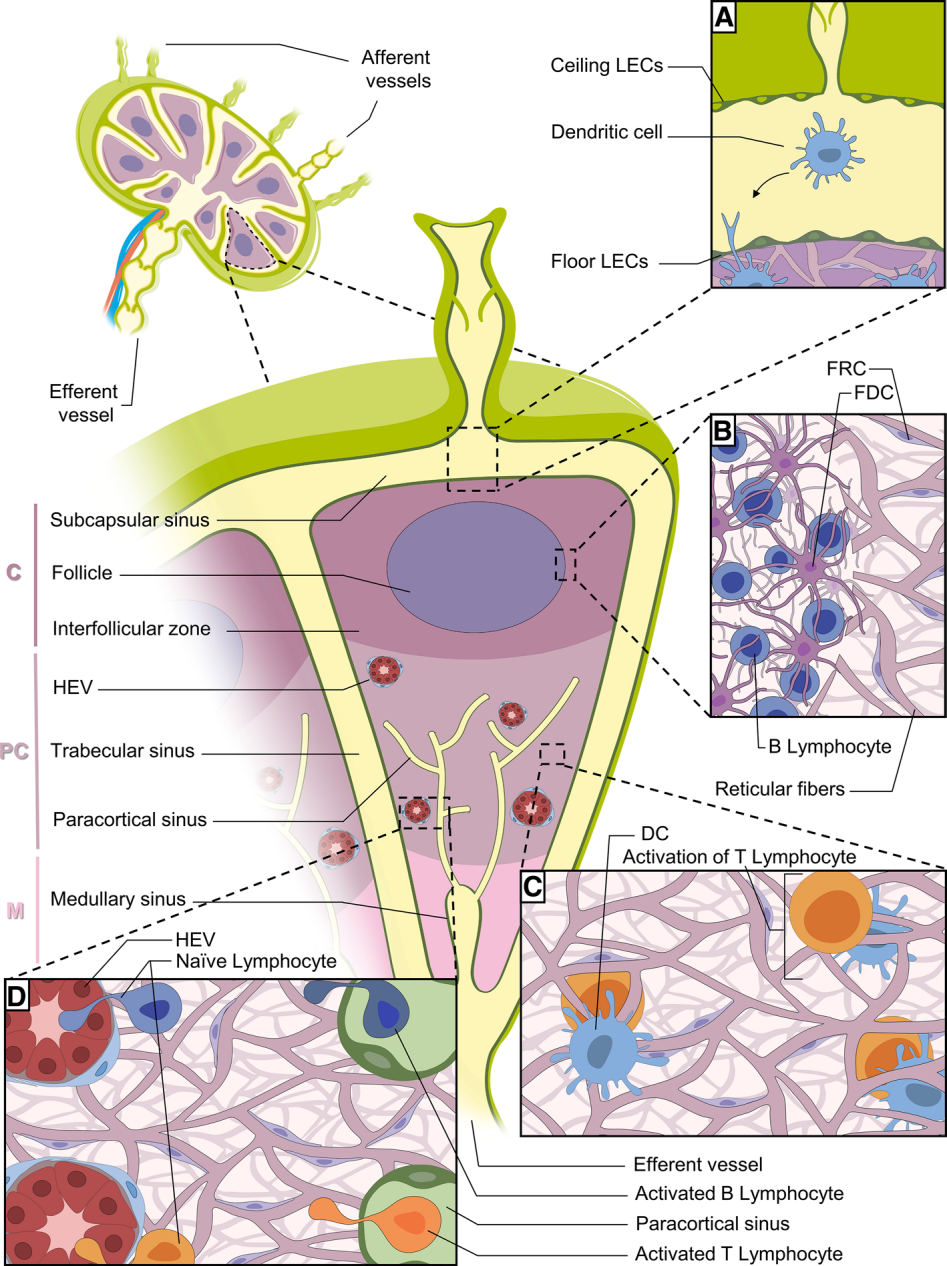
Lymph node (LN) organization. The LN is divided into three parts: the cortex (C), paracortex (PC) and medulla (M). **A** Dendritic cells (DCs) from all over the body arrive at the LN via afferent vessels and then migrate into the cortex (C). **B** B lymphocytes are located in germinal follicles and interact with follicular dendritic cells (FDCs). **C** T lymphocytes are in the paracortex to interact with DCs. **D** DCs migrate on reticular fibers to the high endothelial venules (HEVs), where they interact with naïve lymphocytes entering the LN from the HEV. Activated B and T lymphocytes crawl along the medullary sinus to leave the LN

LNs are immune organs occupying strategic positions throughout the body. There is a complex network of lymphatic sinuses surrounding a highly organized parenchyma composed of reticular fibers, supporting immune cells, specialized blood vessels and fibroblastic reticular cells (FRCs). FRCs play a key role in B and T cell compartmentalization in LNs and, together, represent between 20 and 50% of the non-hematopoietic component of them. These specialized cells express molecules commonly found in myofibroblasts, including desmin, vimentin, CD90, CD73, CD103, α-smooth muscle actin (αSMA) and the ERTR7 antigen [42]. FRCs form stellate cell–cell contacts, thereby creating a three-dimensional network along which leukocytes can migrate. They also produce fibroreticular fibers which are involved in molecular transportation and cell migration. Recently, heterogeneity of stromal cells has been identified in murine LNs [43]. In fact, a number of subsets were identified, including marginal reticular cells, which produce CXCL13 which has a key role in B cell homing and migration towards follicles [44]. In the paracortex, two divergent subsets have been distinguished and express different levels of CCL19, a regulator of lymphocyte migration [43]. This organization provides an optimal environment for immune response induction and regulation[45]. The LN is divided into three areas: the cortex, paracortex and medulla (Fig. 1). The cortex contains follicular dendritic cells and B cells that are mainly associated with germinal follicles, where follicular dendritic cells present antigens to naïve B lymphocytes, leading to antibody production by activated B cells. An interfollicular zone is also present in the cortex and separates the germinal follicles. The paracortex is known as the T cell zone in which antigen-presenting dendritic cells (DCs) prime naïve T lymphocytes. The medulla contains a complex network of medullary sinuses (MS), which converge at the hilum into the efferent LVs [45, 46]. This region contains blood vessels, antibody-secreting B cells and macrophages, which express markers such as CD169, F4/80, MARCO and CD206 [47, 48].

Recent advances have identified intriguing LEC plasticity, heterogeneity and origin diversity [49, 50]. In LNs, different LEC subtypes have been identified in the different anatomical sites described above in both humans and mice [51–54]. Interestingly, SCS LECs and MS LECs display distinct features, including cellular organization, expression profiles, and roles [51]. Mouse SCS LECs produce macrophage scavenger receptors which are involved in the transmigration of lymphocytes entering LNs from peripheral tissues. MS LECs, which express high levels of PD-L1, can be said to contribute to the deletion of alloreactive CD8 + T cells [55]. In humans, however, an additional subset was identified in the MS and cortical sinuses which expressed the C-type lectin CD209, allowing the adhesion of neutrophils to the medulla. In addition, NT5E + , LYVE1 + and MFAP4 + are the LECs lining the ceiling of the medulla, whereas LECs from lymphatic capillaries express PDPN, LYVE1 and CCL21[54]. A first transcriptomic analysis from mouse LNs has revealed the existence of two intriguing LEC subsets in the SCS that further support a substantial degree of LEC specialization [51]. fLECs of the SCS secrete neutrophil chemoattractant CXCL1-CXCL5, and LECs lining the ceiling (cLECs) express CCRL1, a chemokine receptor, thereby creating a gradient favorable for DC migration [56]. In humans, these 2 LEC subsets can be distinguished by the expression of caveolin-1 (by cLECs), while fLECs express TNFRSF9 [54]. These data demonstrate a specific signature of LECs although this depends on where they are located within the LN.

The lymph enters the node via the afferent LVs, which pierce the capsule and drain into the space underneath, known as the SCS. The lymph contains lymphocytes, antigens and DCs that are scanned by macrophages when it arrives in the SCS [57]. It filters through the trabeculae, cortical sinuses and MS before leaving the LN via the efferent LV [58]. From the SCS, smaller antigens and soluble molecules can access the interfollicular zone and the paracortex via a tubular network composed of specialized reticular fibers deposited by FRCs [59]. These reticular fibers are made up of a collagen core surrounded by microfibrils and a basement membrane [58]. This highly organized and interconnected network of ECM components generates conduits, which rapidly transport soluble molecules deep into the LN parenchyma. These conduits form a real 3D pipeline-like system known to rapidly distribute lymphatic fluid, soluble molecules and antigens deep into the LN parenchyma [60, 61] and have also recently been found to transport even larger molecules, such as immunoglobulins or virions [62]. This mesh-like network is essentially present in the T cell zone but follicles remain sparse. It extends to the paracortex where the HEVs are located, creating a connection between the SCS and these specialized blood vessels [61]. This particular structural micro-anatomy where hematopoietic cells can circulate, survive, and interact, both together, and with their environment, allows the LN to carry out its task of an initial immune response site. During an immune response, FRCs produce CCL19/CCL21, which assists in the directional cell migration of naïve T cells, B cells and DCs expressing CCR7. During homeostasis and in the presence of infection, this chemokine gradient helps lymphocyte homing and mediates interactions between T cells and DCs [63]. The reticular fibers descend from fLECs towards the HEVs, which are post-capillary venules especially suitable for lymphocyte entry into the LN parenchyma [50]. They are surrounded by pericytes embedded in a thick basement membrane [64]. HEV endothelial cells have a cuboidal shape and express general endothelial markers (CD31, CD34, VE-cadherin and VEGFR-2), specific blood endothelial markers (von Willebrand factor and peripheral lymph node addressin (PNAd) and VEGFR1) [65].

### The LN extracellular matrix

The ECM provides structural scaffolding and biochemical support for tissue function and mechanical integrity and regulates the availability of growth factors and cytokines. It is composed of a network of biochemically distinct components, including fibrous proteins, glycoproteins, proteoglycans and matricellular proteins [66]. Although it has always been described as a support structure for tissue architecture, it is, in fact, a highly dynamic compartment that regulates a large number of cell functions. An integral feature of the ECM is that it constantly remodels itself as ECM components are deposited, degraded, or modified by ECM-modifying enzymes such as matrix metalloproteinases (MMP) and lysyl oxidase (LOX). The ECM plays a crucial role, not only in the primary tumor [67] but also in the secondary site, particularly at a pre-metastatic stage [68, 69].

Collagen accounts for the largest number of ECM proteins, but its composition and structure vary across different tissue types [70]. For instance, the basement membrane surrounding endothelial cells mainly consists of collagen type IV, while the fibroreticular stroma is, for the most part, composed of fibrillar types I and III collagen embedded in a meshwork of fibrillin microfibers. In LNs, reticular fibers form the principal ECM fibers which support the lymphoid organ architecture. The reticular arrangement of those fibrils is particularly suited to forming conduits and they transport antigen and signaling molecules, as well as guiding migrating cells [71]. Reticular fibers begin at the SCS and extend to the MS. Fibrillin-1 and -2 are essential matricellular proteins in the LN that connect collagen fibers and the basement membrane in tubular structures [71]. Fibrillins constitute the structural backbone of microfibrils, which are found in many elastic and non-elastic tissues where they carry out a diverse number of functions, including interactions with latent transforming growth factor-binding proteins (LTBP) described below [72].

In the majority of organs, fibroblasts are the main source of ECM components, including at least type I and III collagens, elastin, fibronectin, tenascin (TNC) and periostin (POSTN) [24]. In LNs, on the other hand, FRCs are the primary producers of ECM components [59]. Under physiological conditions, these cells produce fibrillary types I and III collagen, collagen type IV, laminin, fibronectin and TNC, which allow cell migration within the LN [59, 73]. A transcriptional analysis performed on murine LNs confirmed that FRCs expressed integrin subunits such as αV, α4, α5, α6, α9, β1, β3, and β5, enabling their adhesion to many ECM components [74]. For example, integrin α5β1 can bind to fibronectin, and αVβ3 interacts with fibronectin, vitronectin, fibrinogen, thrombospondin and POSTN [75, 76]. TNC can bind to numerous integrins, including α2β1 and ανβ3, but the TNC-integrin α9β1 interaction is considered to be of higher avidity [77].

## Contribution of tumor-secreted EVs to the formation of the pre-metastatic LN niche

Extracellular vesicles (EVs), including exosomes, are released by a range of cells and contain proteins and nucleic acids but are produced in larger quantities by tumor cells than by normal cells [78, 79]. Metastatic cancers produce EVs that are able to prime a pre-metastatic niche. Cancer-derived EVs are thought to be involved in the suppression of innate immune responses through the mobilization of MDSCs and the activation of TAMs and neutrophils [80, 81]. However, the detailed mechanism through which EVs promote the pre-metastatic niche is not yet fully understood. miR-105 is expressed and secreted via EV by metastatic breast cancer cells and can be transferred to endothelial cells. Tumor-secreted miR-105 targets ZO-1, leading to increased vascular permeability and metastasis and has been detected in the blood of tumor-bearing mice in the pre-metastatic stage [82]. Recently, miR-25-3P has been shown to promote pre-metastatic niche formation by enhancing vascular permeability and angiogenesis. Tumor-secreted miR-25-3P can also be transferred to vascular endothelial cells where it targets KLF2 and KLF4. KLF2 inhibits VEGFR-2 promoter activity, and KLF4 regulates the integrity of the endothelial barrier [83]. A prospective study has recently revealed that lymphatic EVs from afferent LVs inhibit DC maturation. Through a proteomic analysis performed on lymphatic exudates from patients with primary melanoma, a signature of 18 immune-modulating proteins was identified, including S100A9, a known inhibitor of DC maturation [5]. These data suggest that EVs present in draining lymphatics contain a panel of molecules capable of inducing pre-metastatic niche formation in melanoma patients. Broggi et al*.* compared lymphatic exudate contents from metastatic melanoma patients to the plasma from all patients [84]. They observed that lymphatic exudate was enriched in melanoma-associated proteins but with a fivefold increase in the numbers of EVs. The proteomic profile of EVs from patients undergoing lymphadenectomy with negative LNs was associated with pathways such as VEGF, integrin and cellular extravasation. On the other hand, in patients undergoing lymphadenectomy with positive LNs for tumor cells, upregulation of proliferation, cancer and cell death pathways was observed. Moreover, the expression of S100 was significantly higher in patients with positive LNs than in patients with non-metastatic LNs [84]. These data suggest that EVs from early or advanced melanoma express protein signatures that correlate with different stages of the metastatic process. Tumor-derived EVs were injected intradermally into transgenic mice lacking dermal lymphatics and were nearly undetectable in tissues compared to WT mice, suggesting that lymphatic vessels are actively involved in the transportation of EVs. Moreover, this demonstrated that LECs were the main stromal cells taking up EVs in the tumor-draining LNs [84]. Similar results were observed by Garcia-Silva et al*.* [85], who also observed that lymphatic exudate had a higher level of S100 protein than plasma. Interestingly, the BRAF^V600E^ mutation was detected in exudate-derived vesicles [85]. All these data suggest that exudate-derived EVs could represent a new prognostic tool for melanoma progression and for detecting melanoma mutations. Moreover, these data support the existence of a pre-metastatic niche and the role of LNs in tumor progression. Further details on EV implications in LN metastatic dissemination, can be found in a recent review [86].

## Vascular remodeling in the pre-metastatic LN niche

Lymphangiogenesis and HEV remodeling are key events in the formation of the LN pre-metastatic niche. LN lymphangiogenesis is mainly driven by VEGF-A, VEGF-C, integrin and erythropoietin and correlates with increased systemic metastasis [8, 27, 28, 87, 88]. Lymphangiogenic factors such as VEGF-C are released in the primary tumor by cancer cells and stromal cells, among which macrophages are an important source [89]. VEGF-C stimulates LEC proliferation and migration, inducing the sprouting of LVs and the enlargement of existing vessels, thereby increasing the potential surface of lymphatic contact with tumor cells [90]. Furthermore, the enlargement of collecting lymphatics due to LEC proliferation and structural remodeling of smooth muscle cells results in an enhanced flow rate and increases sentinel LN metastases [91]. Experimental studies have highlighted lymphovascular remodeling in sentinel LNs [27, 28]. Lymphatic remodeling, controlled by soluble factors drained from the primary tumor, within tumor-draining LNs was found to occur even before tumor cells were detected in the LN. It has been suggested that the expanded lymphatic network in LNs contribute to a pre-metastatic niche that promotes LN colonization by metastatic cells [90]. Pre-metastatic induction of lymphangiogenesis in LNs has already been described at length in experimental models. RNA sequencing analysis revealed an altered transcriptional profile of LECs issued from tumor-draining LNs compared to naïve LNs. Interestingly, one of the strongest upregulated genes was integrin αIIb [92], whose expression on a specific subset of LN LECs responsive to RANKL has previously been reported [93]. This integrin, which is upregulated in LECs issued from tumor-draining LNs, promotes LN LEC adhesion to fibrinogen. Another integrin, crucial for LN colonization by tumor cells, such as melanoma cells, is integrin α4. The activation of this integrin is increased by VEGF-C and the PI3Kα signaling pathway and promotes the expansion of the lymphatic endothelium in LNs. This activation also serves as an adhesive ligand that captures VCAM-1 + metastatic tumor cells, thereby promoting LN metastasis [87]. VCAM-1 is also upregulated in tumor-associated LECs and, importantly, increases lymphatic permeability by weakening lymphatic junctions through a mechanism triggered by its interaction with integrin α4β1 [94].

Single-cell RNA sequencing of LECs isolated from naïve murine LNs was performed by Fujimoto et al. [52]. Four subsets of LECs were identified, corresponding to distinct anatomical locations. cLECs were negative for LYVE1 and ITGA2B but positive for CCRL1 (chemokine receptors) and FLRT2, all of which play a role in cell–cell adhesion. Conversely, fLECs expressed LYVE1, ITGA2B and MADCAM but not CCRL1. The expression of genes coding for cell adhesion, such as MADCAM, ITGA2B and FLRT2, suggested that fLECs and cLECs could be the first LECs encountered by tumor cells, allowing LN colonization. Due to the expression of chemokines and chemokine receptors, fLECs and cLECs could also play a role in tumor cell migration [53], although there is currently no clear evidence for the implication of cLECs and fLECs in tumor progression. However, the increased ITGA2B expression in LN LECs during tumorigenesis suggests its involvement through mechanisms yet to be explained [92]. Two other LEC subsets were identified. The first, medullary LECs, defined by the expression of markers such as MRC1 and MARCO. The second subset was cortex LECs expressed unique markers, including PTX3, ITIH5 and KCNJ8. In addition, a specific cortical LEC subtype implicated in rapid lymphocyte egress from LNs was identified [52]. In parallel, another study conducted on murine LN samples provided similar results but defined eight populations of LECs, including the four subsets described above and four new populations, including collecting valve LECs, a bridge population (between cLECs and fLECs) and transition zone LECs (tzLECs) [53]. It is worth noting that no specific gene markers of tzLECs were identified and only a variable expression of MADCAM, CCL20, MARCO and LYVE1. In this study, a clear distinction was made between medullary LECs by the expression of MARCO-LECs and CD274 + and PTX3-LECs (CD274-), but there was no distinction of cortex LECs [53]. Transcriptional profiling of LECs isolated from the LNs of mice bearing tumors has been reported by Commerford et al*.* [92]. Takeda and colleagues have recently conducted a single-cell sequencing analysis of non-sentinel LN LECs (distant from the tumor) collected from cancer patients [54]. In line with the mouse data, SCS cLECs, SCS fLECs and medullary sinus LECs were again distinguished. Two additional subsets were identified for lymphatic valves and capillary lymphatics. Blood endothelial cell heterogeneity in naïve murine LNs was also addressed. Eight different subtypes of blood endothelial cells from mouse LNs were identified with different gene expression. They included arterial ECs, two venous subsets, five capillary subsets, high endothelial cells (HECs) and non-HEC veins, and HECs express genes required for lymphocyte recruitment, such as Glycam1 and Chst4 [95]. Despite these important advances, there remains a need to discover how these lymphatic and blood endothelial subtypes contribute to the pre-metastatic LN niche.

The remodeling of HEVs in tumor-draining LNs is likely to impair the recruitment of naïve lymphocytes and the anti-tumor immune response and may also increase the supply of oxygen and nutrients to a growing metastatic lesion [8]. The features of these blood vessels can again be altered by the primary tumor, even before the appearance of metastases. These alterations are characterized by the dilation and flattening of the endothelium as well as a loss of functional molecules prior to colonization by tumor cells [29, 96, 97]. Bone morphogenetic protein-4 (BMP-4) expression is reduced in HEVs of tumor-draining LNs. This decrease in BMP-4 is implicated in HEV morphology by changing the shape of endothelial cells from a cuboidal to a flattened shape [98]. HEV remodeling further contributes to tumor-induced immunosuppression by interfering with lymphocyte trafficking. To study the role of HEVs in tumor dissemination, Brown et al. [16] developed a model of intralymphatic injection to directly add a number of fluorescent tumor cells into the LN SCS. To determine the importance of HEVs, the efferent LVs were ligated to avoid lymphatic dissemination. Eleven days after injecting the tumor, mice developed lung metastases. Tumor cells became progressively associated with HEVs during tumor progression and frequently localized in their lumen. This experimental study provided evidence that HEVs represent an escape pathway for tumor cells to exit LNs and spread to distant organs using blood circulation [16, 17]. Structural and molecular remodeling of HEVs has been more recently observed in patients with breast cancer although not in healthy patients. This remodeling was associated with the dysregulation of CCL21 in perivascular FRCs, disturbing the migration of CCR7 + naïve lymphocytes in the LN parenchyma [99].

## Immunosuppressive microenvironment in pre-metastatic LNs

**Fig. 2:**
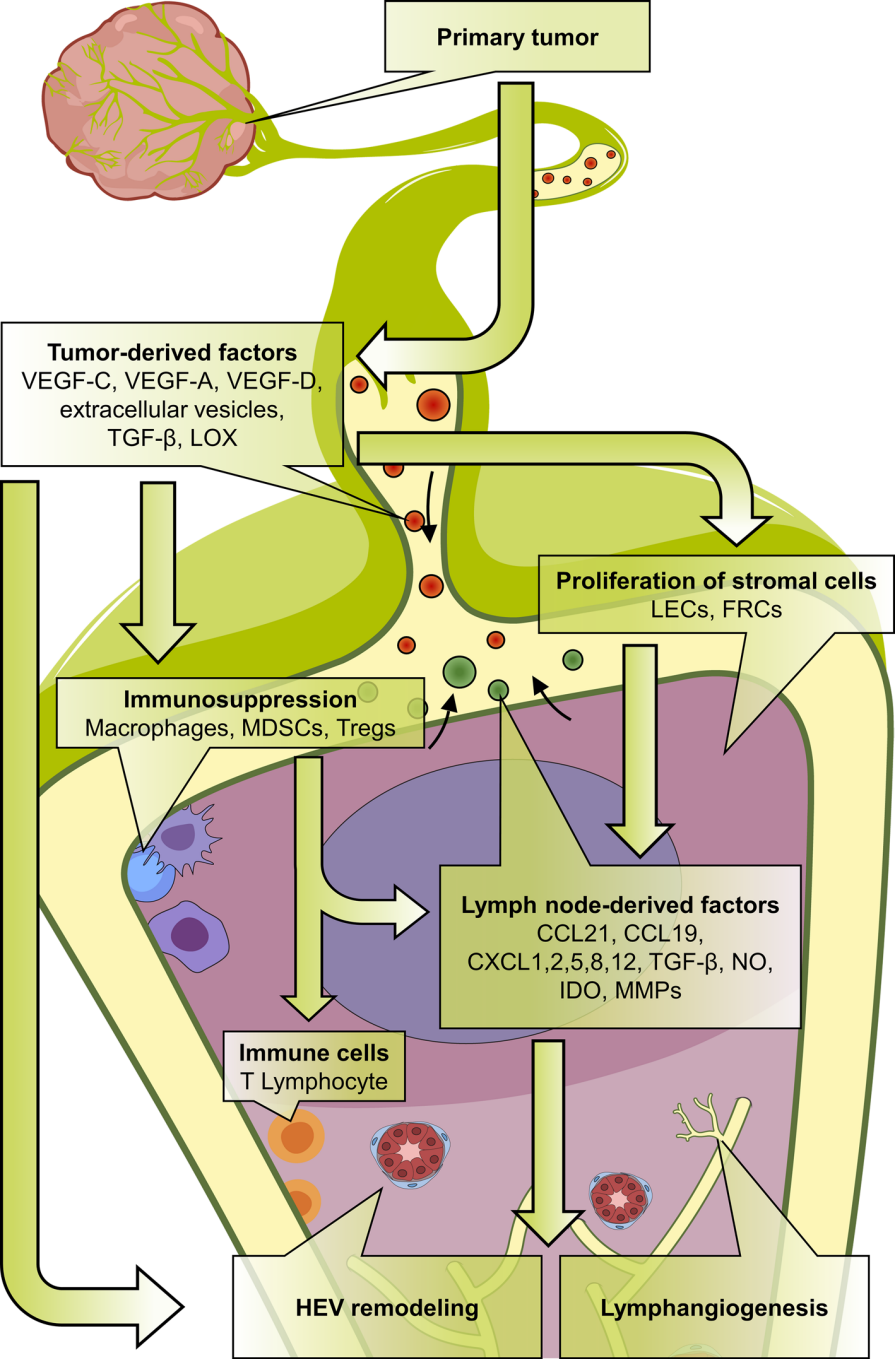
Establishment of the lymph node (LN) pre-metastatic niche. Tumor-derived factors, including vascular endothelial growth factor (VEGF-A, VEGF-C and VEGF-D), extracellular vesicles, TGF-β and lysyl oxidase (LOX), induce an immunosuppressive microenvironment by recruiting macrophages, myeloid-derived suppressor cells (MDSCs) and regulatory T cells (Tregs). Proliferation of lymphatic endothelial cells (LECs) and fibroblastic reticular cells (FRCs) drives the production of LN factors such as chemokines (CCL19; CCL21; CXCL1, 2, 5, 8, and 12); TGF-β; matrix metalloproteinases (MMPs); indoleamine-2,3-dioxygenase (IDO); and nitric oxide (NO), which induce high endothelial venule (HEV) remodeling, stimulate lymphangiogenesis, and regulate tumor cells chemoattraction at metastatic stage

The LN is a dynamic organ subjected to important remodeling at histological, cellular and molecular levels under pathological conditions. In the context of cancer, it is believed that tumor antigens can induce an anti-tumoral response in LNs that initially restricts metastasis formation. Nevertheless, as tumors develop, immunomodulatory factors, drained from the tumor, prime an immunosuppressive response in the LNs that supports metastatic outgrowth (Fig. 2) [10]. Several immune cells, such as myeloid-derived suppressor cells (MDSCs), tumor-associated macrophages (TAMs), Tregs and immature DCs, all play a central role in tumor growth and metastasis and by accumulating in LNs can inhibit the anti-tumor immune activities of CD4, CD8 T cells and NK cells [100–102]. MDSCs are precursors of macrophages, DCs, granulocytes and myeloid cells and are key actors in eliciting immunosuppression. Myeloid differentiation and MDSC expansion are promoted by a variety of molecules, such as GM-CSF, M-CSF, IL-3, IL-6 and VEGF which are produced by tumor cells [103] and the mechanisms used to recruit MDSCs in tumor-draining and distant LNs are described[100, 103–107]. Immunosuppressive activity exerted by MDSCs involves several mechanisms acting on distinct targets through a consistent panel of molecules, including arginase 1, indoleamine-2,3-dioxygenase (IDO), NOS, ROS, peroxynitrite, TGF-β and IL-10 [108, 109]. IDO is an enzyme metabolizing tryptophan that can be expressed by a number of different cell types including DCs. IDO decreases the immune response of T cells and is likely to play a role in the establishment of an immunosuppressive microenvironment in LNs [110]. In fact, a correlation has already been established between the co-expression of IFN-γ and IL-10 and the expression of IDO in sentinel LNs [111]. The function and fate of MDSCs are dependent on their living environment. In lymphoid organs, high STAT3 activity prevents their differentiation into dendritic cells and macrophages and therefore induces their accumulation [112]. The principal target of MDSCs is the T lymphocyte compartment, a deficiency of which is associated with a poor prognosis [113] and targeting essential amino acids is an immunosuppressive strategy used by them [114]. Upregulation of arginase 1 activity leads to the depletion of L-arginine, which is essential for T cell proliferation [115]. MDSCs are also responsible for cysteine depletion and in the microenvironment this was found to impair T cell activation [116]. By secreting IDO, MDSCs also decrease the level of tryptophan, leading to T cell apoptosis via kynurenine generation [117]. The production of NO, which reacts with superoxide, promotes the production of peroxynitrite by MDSCs and this can cause nitration and nitrosylation of the T cell receptor, leading to T cell tolerance [118]. By nitrating chemokines such as CCL2, peroxynitrite also impairs T cell migration [119] although TGF-β and IL-10 represent the main immunosuppressive MDSC-derived factors owing to their ability to inhibit cytotoxic activity and T cell activation [108]. As a result of the expression of PD-L1 and FAS-L, binding the respective ligands PD-1 and FAS present at the T cell membrane, MDSCs also exert immunosuppressive activity through direct contact with T cells [120, 121] and can also induce the expansion of Tregs, another major immunosuppressive actor [122, 123]. These features highlight the dual role of recruited MDSCs in permissive microenvironment generation. Indeed, they are directly responsible for two synergic and complementary processes, immunosuppression and immunotolerance, which make them attractive therapeutic targets to overcome cancer immune escape strategies [124].

Together, these immune cells actively contribute to the formation of the pre-metastatic niche, necessary for LN colonization by metastatic cells that can eventually exit from the LN into the blood circulation [101, 125]. They modulate the local microenvironment by secreting inflammatory cytokines, growth factors, pro-angiogenic molecules and enzymes that remodel the matrix, such as LOX and MMPs [126].

Macrophages are present throughout the LN but are classified in different subtypes according to their location. A distinction is made between macrophages present in the SCS and MS from those residing in the LN parenchyma [47]. SCS macrophages are able to capture antigens from the lymph and transfer them to B cell follicles, but they appear poorly phagocytic. In contrast, CD209 + MS macrophages are more phagocytic and express F4/80. Both types are characterized by CD169 expression, a member of the sialic acid-binding lectin family. [48]. LECs play an important role in the maintenance of these macrophages via RANKL production and they are lost when there is RANKL deficiency [127]. LECs produce CSF-1 and this also plays a crucial role in the maintenance of the macrophages, as well as the MS macrophages [128]. An additional type of macrophage present in the LN germinal center is tangible body macrophages, which have a particular role in the uptake of apoptotic cells within germinal centers [48]. Macrophages are also present in the parenchyma adjacent to the MS known as the medullary cords [47]. The last subset of parenchymal macrophages resides in the T cell zone. They express CD11c, CX3CR1, CD64 and MER proto-oncogene tyrosine kinase (MERTK) but test negative for CD169 and F4/80 [129]. Modifications in the CD169 + macrophage density have also been reported in pre-metastatic LNs. These macrophages capture tumor-derived antigens in the SCS and transfer them to CD8 + T cells to elicit an anti-tumor response and can also capture EVs derived from tumor cells [86]. In a pre-clinical model, mice lacking CD169 + macrophages failed to induce anti-tumor immunity [130]. Reduced CD169 expression in pre-metastatic LNs is associated with subsequent metastatic disease and a poor outcome in several tumor types [131–134]. Tumor-derived EVs bind SCS CD169 + macrophages in tumor-draining LNs [135]. These macrophages are a major host cell type interacting with EVs in tumor-bearing mice. 3D imaging of tumor-derived LNs with decreased CD169 + macrophages showed a higher penetration of EVs in the LN cortex. These data therefore suggest that SCS macrophages act as EV scavengers in an attempt to prevent cancer progression [135]. In humans, the presence of macrophages testing positive for HMB-45, a transmembrane glycoprotein expressed by melanomas, was localized near the LN capsule. LNs proved negative for tumor cells, suggesting that tumor-derived factors reach LNs in cancer progression, supporting the hypothesis of the pre-metastatic niche [135]. Prostaglandin E2 (PGE2) also plays an important role in the LN pre-metastatic niche and has been identified as an immunosuppressive molecule that increases the immunosuppressive potential of Tregs. PGE2 can also stimulate the expression of CXCL12 via the EP3 receptor, which increases the accumulation of CXCR4 + tumor cells and promotes the formation of the LN pre-metastatic niche [136].

Beyond the vessel wall lining functions described above, LECs can play a key role in immunosuppression, facilitating metastatic cell survival. LECs express inhibitory ligands such as PD-L1, which allows CD8 lymphocyte suppression or deletion [137]. LN LECs can also cross-present tumor antigens to promote CD4 suppression and produce immunosuppressive molecules such as nitric oxide, TGF-β, and IDO to promote an immunosuppressive nodal microenvironment [137–142]. It is known that both MHC class I and MHC class II are present in LN LECs [84, 143], and play an important part in immunotolerance and immune response. MHC I plays a crucial role in self-tolerance by presenting endogenous antigens to CD8 + T cells. Tumor-draining LN LECs were able to cross-present tumor antigens using MHC I and directly alter the CD8 + T cell response [9, 143, 144]. In addition, through acquiring MHC II from DCs, LECs were also shown to induce CD4 + T cell tolerance [145, 146]. fLECs can also be distinguished from other LEC subsets due to the expression of CD74 which is involved in the formation and transport of MHC class II antigen complexes [53].

The most striking TGF-β function is immunosuppression, of paramount importance in the context of cancer. Indeed, TGF-β is able to induce the expression of cell cycle regulators (p21 and p27), which inhibit the proliferation of naïve T lymphocytes [147]. TGF-β inhibits antigen presentation of DCs by suppressing the expression of major histocompatibility complex II [148] and promotes the differentiation of T cells in Tregs by triggering the expression of FOXP3 [149]. The emerging picture is that latent TGF-β could be activated through two different mechanisms, one involving LTBPs associated with the ECM and the other implicating transmembrane glycoprotein A repetitions predominant (GARP) [72, 150, 151]. Both mechanisms of TGF-β involve an integrin, binding to LAP to induce its mechanical deformation and the release of mature protein. The role of GARP has been mainly studied in Tregs, although it can be produced by non-immune cells such as endothelial cells and fibroblasts [152].

Under physiological conditions, TGF-β1 is the predominantly expressed isoform in immune cells, including immunosuppressive Tregs. Immunosuppression by the TGF-β1 pathway through Tregs avoids autoimmune reactions but contributes to tumor development [153]. Furthermore, myeloid cells such as TAMs, MDSCs and tumor-associated neutrophils also promote tumor progression by elaborating a pre-metastatic niche through an increased production of TGF-β [154].

The role of TGF-β in immunomodulation in LNs has been less well documented. Huang et al. demonstrated in a mouse model that Tregs secrete TGF-β1 in LNs [155], which in turn induces the expression of IL-17rb in 4T1 cells via the Smad2/3 signaling pathway boosting tumor malignancy [155]. Furthermore, the integrin-mediated regulation of TGF-β activation is essential for naïve T cell conditioning by DCs in LNs [156]. Interestingly, αvβ8 integrin-deficient mice, either globally or specifically in DCs, spontaneously develop severe immune cell deficiencies due to the impairment of TGF-β1 activation [157]. Further work is required to determine the exact contribution of TGF-β and its regulators (LTBP, GARP, integrins) to pre-metastatic LN niche formation.

## LN extracellular matrix remodeling in the pre-metastatic niche

While cancer-associated fibroblasts (CAFs) represent a major cellular component of most primary neoplasms [158], these cells have only been poorly described in metastatic organs, particularly in LNs to date. Interestingly, a recent study identified four CAF subtypes in metastatic LNs of breast cancer patients [159]. Two of these subtypes (CAF subtype 1 and subtype 4) produce TGF-β and CXCL12 and activate the NOTCH signaling pathway to promote tumor cell invasion. Intriguingly, the origin of those CAFs in LNs remains unclear [159, 160]. Additional studies are required to increase knowledge of putative CAF implications in pre-metastatic and metastatic LN niches.

ECM remodeling is a key event that contributes to metastatic organ pre-conditioning and to the formation of an appropriate environment for tumor seeding. ECM modifications in the pre-metastatic niche have already been described, in detail, for the lung, liver and bones but have been poorly documented in the case of LNs. Interestingly, organ specificities have been highlighted in terms of ECM remodeling [24]. Among the ECM proteins involved in the metastatic colonization of distant organs (lung, liver, and bone) are TNC, POSTN and versican, the large chondroitin sulfate proteoglycan, which have been identified as key players [26, 161–163]. POSTN plays a major role in tissue remodeling by interacting with ECM proteins such as fibronectin, TNC and collagen types I, IV, V [164]. POSTN-knockout mice bearing breast tumors exhibit decreased MDSC accumulation in pre-metastatic lungs and percentages of CD4^+^ and CD8^+^ T cells were more prevalent in the lung, and immunosuppressive factors were reduced in them compared to levels in wild-type mice [104]. POSTN is thought to play its part in LN metastasis but has not been clearly demonstrated at the present time. Recently, POSTN has been identified in metastatic LNs from patients with cervical cancer [165]. CAFs expressing POSTN, impaired lymphatic integrity by activating the integrin-FAK/Src-VE-cadherin signaling pathway in LECs, thereby increasing metastatic dissemination. Interestingly, CAF-derived POSTN was not found in non-metastatic LNs, suggesting the importance of the role of POSTN in tumor cell dissemination [165]. Unfortunately, no evidence has been provided about POSTN in the LN pre-metastatic niche, and this needs further study. Increased fibrinogen deposition was found in tumor-draining LNs compared to control LNs [92]. Furthermore, enhanced production of ECM-remodeling factors such as LOX, MT1-MMP and TIMP-1 was detected in metastatic LNs from patients with oral cancer [166]. This was in line with the implication of LOX and MMPs in the liver and lung pre-metastatic niches [167–170]. For instance, MMP9 induced by primary tumors in lung endothelial cells and macrophages promotes the invasion of tumor cells into the lung [171]. MDSCs recruited in the lung are also an important source of MMP9 [172]. LOX can also promote the production of MMP9 and fibronectin by fibroblasts in the lung pre-metastatic niche [173]. Taken as a whole, these data highlight important matrix remodeling in LNs at different stages of the metastatic cascade. Additional studies are, however, still required to reveal the role of ECM-remodeling factors in the LN pre-metastatic niche.

## Conclusions and perspectives

A pre-metastatic niche is now widely accepted as a specific tumor-induced microenvironment, favorable for disseminating tumor cells and metastasis formation [170]. The elaboration of a pre-metastatic niche before colonization by tumor cells is a complex process recognized as an initial key step in the metastatic cascade. Recent advances in this field have identified a panel of crucial molecular and cellular components contributing to pre-metastatic niche formation in various tumor models. Factors produced by primary tumors can potentially condition not only the LN microenvironment but also other distant organs, including the lung, liver, brain and bone [174]. The exposure of LNs to a higher concentration of tumor-secreted factors drained by the lymph compared to other organs could explain, at least partially, the predominance of LN metastases in cancer prognosis and in metastatic dissemination [10]. The description of LN lymphangiogenesis and its role in the metastatic process is relatively recent. Only a small number of clinical studies to date have documented pre-metastatic lymphangiogenic variations in the sentinel LNs of patients with cervical, breast, lung and oral squamous carcinomas [2, 97, 175–177]. These studies supported the concept of the LN pre-metastatic niche and revealed that LV density was increased in pre-metastatic sentinels in comparison with non-sentinel LNs. Notably, LV remodeling is also associated with modifications in the immune landscape [2]. Therefore, LN lymphangiogenesis is viewed as a potential target to treat or prevent metastatic disease. In this context, and given the crucial role of the VEGF-C/VEGFR-3 signaling pathway in lymphangiogenesis, a majority of studies aim to develop therapeutic drugs targeting this pathway. A phase 1 clinical trial evaluated an antibody directed against VEGFR-3. Unfortunately, disease control was only observed for a small percentage of patients (19%) [178]. A lack of response of LN metastasis to treatment with inhibitors of VEGFR-2 and VEGFR-3 has also been shown in mouse models [179], but, to date, both pre-clinical and clinical data have failed to demonstrate the efficacy of VEGFR inhibitors on LN metastases. These data emphasize the importance of searching for other putative therapeutic targets. Another clinical trial has used VGX-100, a VEGF-C neutralizing antibody, but no data have been published yet (NCT01514123). Recently, simvastatin, a 3-hydroxy-3-methylglutaryl coenzyme A (HMG-CoA) reductase inhibitor, was tested for LN metastasis in mice. Its interest relies on its capacity to decrease inflammatory cytokine synthesis and circulating VEGF levels. Simvastatin appears to play a potential role in tumor lymphangiogenesis and LN metastasis, suggesting that its combination with other agents could reduce LN lymphangiogenesis and tumor progression [180]. As highlighted in this review, LN pre-metastatic niche formation not only relies on LN lymphangiogenesis but also results from important, but still poorly documented, remodeling of different cellular and matrix components. Therefore, it is probable that a narrow focus on a unique biological process such as lymphangiogenesis or one of these molecular pathways will be unsuccessful for therapeutic development.

A large number of questions remain unanswered. What are the dynamics of LN pre-metastatic formation? It is largely unknown how different ECM components interact in the pre-metastatic niche and exert cooperative/synergistic or antagonistic effects on metastatic tumor cells. Which markers or signatures could be used to stratify patients and/or predict their potential to form LN and distant metastases? Understanding how and when the key cross-talk between the primary tumor and LN is established to prime the organ is a prerequisite to identify the best potential molecular target(s). Consequently, it is crucial that basic scientists and clinicians work together to explore all facets of the pre-metastatic LN niche for diagnostic, prognostic and therapeutic purposes.
